# The Anti-Vivisectionists Again

**Published:** 1901-12

**Authors:** 


### EDITORIAL NOTES AND COMMENTS.

The Business office of THE JOURNAL-RECORD is No. 64 Marietta Street.

The Editorial office is 318-319-320 The Prudential.

Address all Business communications to Dr. M. B. Hutchins, Mgr.

Make remittances payable to THE ATLANTA JOURNAL-RECORD OF MEDICINE.

On matters pertaining to the Editorial and Original Communications address Dr. Bernard Wolff, Atlanta.

Reprints of original articles will be furnished at cost price. Requests for the same should always be made *on the manuscript.*

We will present, post-paid, on request, to each contributor of an original article, twenty (20) marked copies of THE JOURNAL-RECORD containing such article.

### THE ANTI-VIVISECTIONISTS AGAIN.

WE have been favored with a marked copy of the *Journal of Zoophily,* which word we take to mean life-loving, or a friend to life. Further perusal of the *Journal* arouses the question as to the exact significance of the word, for it favors the life of the animal over that of man. It seems animated by thought that the squeak of the rabbit shall drown out the piteous cry of the child strangled in the malignant grasp of diphtheria, and that a few worthless animals are to be protected more than him who is made in the image of God. It is wonderful that intelligent men and women should fail to see that doctors in conducting experiments upon living animals are in the highest degree humane, and that beside their mighty object the purposes of the anti-vivisectionists wither into colorless insignificance. The principle that runs all through is the appropriation of one animal by another for the latter's good.

"The swallow is pierced by the shrike,
And the swallow chases the bee."

Is it any more brutal and cruel to sustain life by the flesh of animals than to endeavor to utilize it in other directions for the prolongation of life and the alleviation of some of its multitudinous ills? Still comes the coo-coo cry that Lawson Tait said that vivisection (with all included under the word) was unnecessary and useless. Yet there are more little children who will rise up to call Roux and Behring blessed than ever there are spayed women to glorify the name of Lawson Tait.

These are the larger bearings of the question; as concerns the lesser, it is freely admitted that some doctors are rash, some make mistakes from scientific zeal, some are simply cruel, but to impute cruelty to every man of science who performs experiments upon living animals is singularly absurd. Pity is always present and is manifested in the almost invariable use of anaesthesia, which is furthermore rendered necessary to facilitate the demonstration—

> "Pity thee? So I do,
> I pity the dumb victim at the altar;
> But does the robed priest for his pity falter?"

The truth is that the anti-vivisectionists are nothing more nor less than obstructionists, who interpose themselves before the march of progress and are destined to be crushed under the wheels. They should leave the question of cruelty to be settled by those who are far more intelligent, far better able to judge than themselves, and cease their idle bickering and vaporing.

